# Characteristics and consequences of falls, and preventive interventions in hospitalised patients: A cohort study

**DOI:** 10.1371/journal.pone.0358336

**Published:** 2026-11-13

**Authors:** Maria Unbeck, Eila Sterner, Åsa Gransjön Craftman, Helene Andersson-Forsström, Louise Egberg

**Affiliations:** 1 School of Health and Welfare, Dalarna University, Falun, Sweden; 2 Department of Clinical Sciences, Danderyd Hospital, Karolinska Institutet, Stockholm, Sweden; 3 Department of Molecular Medicine and Surgery, Karolinska Institutet, Solna, Sweden; 4 Department of Trauma, Acute Surgery and Orthopaedics, Karolinska University Hospital, Solna, Sweden; 5 Department of Neurobiology, Care Sciences and Society, Division of Nursing, Karolinska Institutet, Huddinge, Sweden; 6 Department of Infection, Danderyd Hospital, Stockholm, Sweden; 7 Department of Surgery and Urology, Danderyd Hospital, Stockholm, Sweden; Kyung Hee University, KOREA, REPUBLIC OF

## Abstract

**Introduction:**

In-hospital falls are common, particularly among elderly patients. These falls can result in physical, psychological, social, and financial negative outcomes. The aim was to explore fall characteristics, physical consequences, and preventive interventions in hospitalised adult patients.

**Methods:**

At a teaching hospital, 31,571 episodes of care constituted the study population. A stratified sampling method with five groups was used. The study cohort consisted of 1,232 episodes of care; each reviewed in the electronic patient record using a study-specific protocol.

**Results:**

After adjusting for the sampling technique, the cumulative incidence of falls in the study population was 1.9%. Furthermore, falls per 100 episodes of care and per 1,000 patient days were 2.3 and 4.9, respectively. In 590 episodes of care included in the study cohort, 714 (minimum-maximum 1–7) falls were identified. Two thirds of fallers were identified on admission as at risk. Fallers were often male (p = 0.041), older (p < 0.001), admitted acutely (p = 0.01), had a longer length of stay (p < 0.001), and were admitted to a medical specialty (p = 0.001). Falls were more frequent on weekdays (12.9%–17.1%) and during night shifts (37.7%). The most common location was the patient’s room (48.0%), and the most common position was when walking upright (28.9%). Of patients assessed as fall-risk individuals at admission, 58.2% had ≥ 1 documented fall-preventive intervention. In total, 1,933 interventions were prescribed, most commonly environmental adaptations, personal assistance during mobilisation, and use of non-slip socks or shoes. Most falls (60.1%) caused no harm; injuries were usually minor, including pain, wounds, and hematoma/swelling. Median time to first fall was 2 days (95% confidence interval 1.0–2.0). Elective patients fell earlier than acute patients.

**Conclusion:**

Understanding falls can increase knowledge and advance care. Despite fall-preventive interventions, falls persists. Personalised, evidence-based interventions, particularly early in hospitalisation and at night, may reduce falls and improve outcomes.

## Introduction

In 2021, the World Health Organization launched a global patient safety action plan to promote coordinated, systemic changes aimed at eliminating preventable [1]. Although patient safety has improved in many areas of healthcare and progress has been made in numerous countries, significant challenges remain. It is estimated that more than 10% of patients are harmed in healthcare, and majority of these adverse events are deemed preventable [2].

One example of an adverse event is in-hospital falls. The World Health Organization defines a fall “as an event which results in a person coming to rest inadvertently on the ground or floor or other lower level” [3]. In-hospital fall rates vary from 0.41 [4] to 16.9 [5] falls per 1,000 patient days and contribute to a significant proportion of preventable harm [1].

The severity of a fall varies widely from no harm to the death of the patient. Up to 72.1% of the falls involves harm [4]. The impact of falls, regardless of whether harm occurs or not, may affect a patient's overall health, quality of life and recovery [6]. Falls are linked to physical negative outcomes such as limited mobility, but also to physiological and social consequences, including fear of future falls, a sense of being a burden to others, and decreased social engagement [7].

Falls during hospitalisation can lead to, for example, increased length of stay and additional treatments and care, contributing to a substantial financial burden on the healthcare systems, other services, and patients/families. An Australian study [8] reported that, apart from increased costs, falls led to longer hospitalisation, with an average stay of 8 days. Dykes et al. [9] found minimal cost differences between harmful and non-harmful falls and demonstrated that an evidence-based fall prevention program reduced both fall incidence and related costs.

Despite decades of research, ongoing clinical efforts, and guidelines, hospital falls remain a significant risk to patients and are a subpart of the global patient safety challenges [10]. Knowledge about the characteristics and consequences of in-hospital falls is important to improve fall prevention strategies and ensure patient safety. Although there is an increasing body of evidence addressing various aspects of the complex multifactorial nature of these falls, further knowledge is required. There is a need to provide more detailed knowledge regarding falls, such as occurrence, location, position, fall-related activities, fall-preventive interventions, severity, type of injury, actions taken and timing of falls with or without injury. This study adds to the literature regarding these details.

The aim was to explore fall characteristics, physical consequences, and preventive interventions in hospitalised adult patients.

## Methods

### Design

A cohort study based on structured retrospective record review.

### Setting

The study was conducted at a teaching hospital with a capacity of approximately 420 beds. The hospital includes medical and surgical wards, an intensive care unit, and a delivery ward. In 2016, the facility provided care for around 48,000 in-patient episodes and served a catchment area with a population of about 500,000 residents.

### Definitions

The Swedish fall definition [11], based on the World Health Organization, was used: “a fall is an event when a person unintentionally ends up on the floor or ground, irrespectively if an injury occurs or not.”

An episode of care refers to the period from admission to a ward until discharge from that ward, excluding time spent in the Emergency Department.

### Hospital guidelines for fall prevention

According to the hospital guidelines for fall prevention, risk assessment of all patients, within 24 hours of admission, using Downton Fall Risk Index (DFRI) was mandatory, regardless of age. Only the specialities of Obstetrics and Gynaecology were exempted from these guidelines. DFRI is also recommended as the first choice on a national level.

The DFRI consists of five nominal modules, representing 11 different risk factors: known previous falls (0–1 point), medications (none, sedatives/hypnotics/neuroleptics, sedatives, diuretics, antihypertensives [other than diuretics], anti-Parkinson drugs, antidepressants, 0–5 points), sensory impairment (none, visual, hearing and motor skills, 0–3 points), cognitive impairment (orientation to person, place, and time, 0–1 point) and walking ability (gait, 0–1 point). These are summarised into a total index score with a range of 0–11. A score of three or greater indicates an increased risk of falling [12].

Registered nurses assess fall risk using the DFRI and clinical judgment. Re-assessment is required if the patient’s condition deteriorates. A pre-defined and adjustable fall prevention plan in the electronic patient record supports documentation with diagnoses, goals, and intervention options. The plan should be established within 24 hours of admission and based on risk factor analysis, ideally in collaboration with the patient. It must be evaluated regularly if the patient’s condition changes.

### Inclusion criteria and sampling strategy

All episodes of care with a discharge date in 2016 (n = 31,571) from the following departments were included in the study population: Cardiology, Infection, Medical, Orthopaedics, Rehabilitation medicine, Renal medicine, and Surgical/Urological. A stratified sampling method was used to divide the study cohort into five groups, two with random sampling and three with total sampling. Details regarding the sampling strategy and data collection are reported elsewhere [13].

### Data collection

A study-specific protocol was developed to standardise the reviews. Trained and experienced reviewers read all health professionals’ entries in the electronic record (TakeCare^©^) related to the sampled episode of care. Data collected included fall occurrence, DFRI use and outcomes, fall-preventive interventions, fall details (timing, location, position, activity), pre-fall anxiety/confusion, harm type, response actions, and severity. The severity of the falls was assessed using a modified version of the National Coordinating Council for Medication Error Reporting and Prevention scale (NCC MERP) [14] (categories C–I). The scale is commonly used both nationally and internationally in retrospective record reviews. In this scale, categories C–D included falls with no harm and categories E–I included falls with harm. Furthermore, patient demographics were obtained from the patient administrative system.

### Data Analysis

Descriptive statistics, including frequencies, percentages, medians (min-max) and means (standard deviation [SD]) were used for analysis. Falls per 100 episodes of care ([numbers of falls/numbers of episodes of care] * 100) and 1,000 patient days ([number of falls/numbers of patient days] * 1,000) were calculated. The length of hospital stay was calculated as (date of discharge – date of admission] +1) and time to first fall was calculated as ([date of fall – date of admission] +1). Chi-square test was used to examine differences in categorical variables between fall and no-fall patients, while independent t-test was applied for continuous variables such as age and length of stay. Due to a high proportion of missing data for some fall demographic variables, sensitivity analyses were conducted. Variables representing missing cases were created for each group (location, position, another person present and fall-related activity). These variables were tested against patient demographics and DFRI outcomes. For continuous variables, the Wilcoxon rank sum test was used, while for categorical variables, the chi-square test was applied. A p-value <0.05 considered to be statistically significant. The median time with 95% confidence interval (CI) until a first fall, the event in calculations, was estimated by the Kaplan–Meier method. Patients without any fall were censored at the time of discharge from the respective in-patient ward. Seventeen patients were excluded in the analysis due to missing values for either admission type (n = 7) or fall date (n = 10). Differences in time until a first fall between patients with acute and elective admissions were compared using a Cox-regression. IBM SPSS Statistics version 25 (IBM Inc., USA) and R version 4.3.0 were used for the calculations.

### Ethics approval

The study was conducted in accordance with international research standards and was approved by the Regional Ethics Committee of Stockholm (2015/744–31/5). As the study involved retrospective review of patient records without direct patient participation, access was granted by the hospital’s Coordinator for Information Safety in accordance with the ethical approval. Individual informed consent was therefore not required or obtained. The researchers were given access to the data on 1 February 2017. The records contained identifiable patient information, which was accessible to the researchers during data collection. Following data collection, the coded dataset could be linked to the patient list.

## Results

### Patient demographics and risk assessment outcomes

Of the 31,571 eligible episodes of care, i.e., the study population (146,972 patient days), 1,232 (3.9%, 10,880 patient days) were included in the stratified sampling and reviewed as the study cohort. No patients meeting inclusion criteria were excluded. The study cohort predominantly consisted of elderly patients and those with acute episodes of care. There were more men than women, and most patients stayed on the ward for less than a week. Most patients were admitted from home and the most common index departments were Medicine and Cardiology. Compared to non-fallers, those patients who suffered a fall were often male (p = 0.041), older (p < 0.001), admitted acutely (p = 0.01), had a longer length of stay (p < 0.001) and were admitted to a medical speciality (p = 0.001) (Table 1).

**Table 1:** Patient demographics, Downton Fall Risk Index (DFRI) outcomes, and registered nurses’ (RNs’) clinical judgments.

Patient demographics	All patients N = 1,232	Patients with no fall n = 642	Patients with ≥ 1 fall n = 590	P-value
Sex, n (%)				0.041
Men	677 (55.0)	335 (52.2)	342 (58.0)	
Women	555 (45.0)	307 (47.8)	248 (42.0)	
Age, median age in years (min-max)				< 0.001
All patients	74 (15-102)	71 (15-102)	79 (18-99)	
Men	74 (15-102)	70 (15-102)	78 (24-98)	
Women	76 (15-99)	72 (15-96)	79 (18-99)	
Age, in two groups, n (%)				< 0.001
15-64	359 (29.1)	241 (37.5)	118 (20.0)	
≥ 65-102	873 (78.9)	401 (62.5)	472 (80.0)	
Type of admission, n (%)				0.01
Acute admission	1,046 (84.9)	526 (81.9)	520 (88.1)	
Elective admission	179 (14.5)	112 (17.5)	67 (11.4)	
Unknown	7 (0.6)	4 (0.6)	3 (0.5)	
Length of stay, median days (min-max)	5 (1-117)	3 (1-100)	7 (1-117)	< 0.001
Admitted from, n (%)				0.241
Home	1,020 (82.8)	539 (84.0)	481 (81.5)	
Another department within the hospital	116 (9.4)	59 (9.2)	57 (9.7)	
Another healthcare provider	77 (6.3)	36 (5.6)	41 (6.9)	
Residential care	16 (1.3)	5 (0.8)	11 (1.9)	
Unknown	3 (0.2)	3 (0.5)	0 (0.0)	
Index department, n (%)				< 0.001
Medicine	408 (33.1)	200 (31.2)	208 (35.3)	
Cardiology	280 (22.7)	159 (24.8)	121 (20.5)	
Surgical and urological	247 (20.0)	141 (22.0)	106 (18.0)	
Orthopaedics	126 (10.2)	79 (12.3)	47 (8.0)	
Infection	69 (5.6)	24 (3.7)	45 (7.6)	
Rehabilitation medicine	51 (4.1)	23 (3.6)	28 (4.7)	
Renal medicine	51 (4.1)	16 (2.5)	35 (5.9)	
Type of speciality, n (%)				0.001
Medical	859 (69.7)	422 (65.7)	437 (74.1)	
Surgical	373 (30.3)	220 (34.3)	153 (25.9)	
Satellite episode of care, n (%)	30 (2.4)	21 (3.3)	9 (1.5)	0.047
**DFRI outcomes and RNs´ conclusive clinical judgments**				
DFRI, assessed at admission, n (%)	1,145 (92.9)	579 (90.2)	566 (95.9)	
DFRI score, mean (SD)/median (min-max)	2.7 (1.9)/3 (0-10)	2.2 (1.8)/2 (0-10)	3.3 (1.7)/3 (0-10)	< 0.001
Fall risk assessed patients with a fall risk according to DFRI (score of ≥ 3), n (%)	619 (54.1)	228 (39.4)	391 (69.1)	
Fall risk according to RNs´ conclusive clinical judgments at admission, n (%)				
Risk of falling	631 (51.2)	229 (35.7)	402 (68.1)	
No risk of falling	535 (43.4)	364 (56.7)	171 (29.0)	
Documentation is missing	66 (5.4)	49 (7.6)	17 (2.9)	

SD, Standard deviation.

Most (92.9%) patients were risk-assessed for falls at admission using DFRI, and of these, 619 (54.1%) were assessed to be individuals at risk of falling according to DFRI. Two thirds of the patients who fell were identified on admission as fall-risk individuals and they had a higher DRFI score (p < 0.001) compared to non-fallers (Table 1).

### Fall incidence

After adjusting for the sampling technique, the cumulative incidence of falls for the study population was 1.9%. Furthermore, the numbers of falls per 100 episodes of care and per 1,000 patient days were 2.3 and 4.9.

Almost half of the patients (n = 590, 47.9%) in the study cohort experienced ≥1 in-hospital fall. The total number of falls was 714, with a range of 1–7 falls per patient who fell. Most of the fallers experienced 1 fall (n = 502, 85.1%), 68 (11.6%) patients had 2 falls, and 20 (3.3%) patients fell 3 or more times.

### Fall demographics

Falls occurred more frequently on weekdays, ranging from 12.9% (Wednesday, n = 92) to 17.1% (Tuesday, n = 122), compared to weekends (Saturday, 11.3%, n = 81; Sunday, 13.2%, n = 94). Falls during night shifts (37.7%) were slightly more common compared to the morning shifts (33.3%). The most common locations for falls were the patient’s room (48.0%) and the bathroom (19.2%). The most frequent position immediately before a fall was an upright position when walking (28.9%). Many falls were unobserved (49.2%). Most falls occurred in connection to bathroom and toilet visits (27.9%), and movement for unknown reasons (13.0%). Falls occurred to the same extent regardless of the documented presence of anxiety/confusion before the fall or not (Table 2).

**Table 2:** Fall demographics for 714 falls.

Variables	
Day of the week, n (%)	
Monday	101 (14.1)
Tuesday	122 (17.1)
Wednesday	92 (12.9)
Thursday	109 (15.3)
Friday	103 (14.4)
Saturday	81 (11.3)
Sunday	94 (13.2)
Not documented	12 (1.7)
Shift, n (%)	
Night shift 22:00–06:59	269 (37.7)
Morning shift 07:00–15:59	238 (33.3)
Evening shift 16:00–21:59	161 (22.5)
Not documented	46 (6.4)
Location, n (%)	
Patient room	343 (48.0)
Bathroom/toilet/shower	137 (19.2)
Common areas at the ward	60 (8.4)
Unspecified location at ward	18 (2.5)
Other locations^a^	8 (1.1)
Not documented	148 (20.7)
Position, n (%)	
From walking	206 (28.9)
From lying down	125 (17.5)
From sitting	120 (16.8)
Not documented	263 (36.8)
Another person present, n (%)	
No	351 (49.2)
Yes	106 (14.8)
Not documented	257 (36.0)
Fall-related activity, n (%)	
In relation to bathroom/toilet visits	199 (27.9)
Movement for unknown reason	93 (13.0)
In the event of a sudden / unexpected change in the patient´s condition	35 (4.9)
When trying to reach own things that should have been within reach	19 (2.7)
In connection with meals	17 (2.4)
In connection with personal hygiene / dressing and undressing	15 (2.1)
Moving from bed to chair or chair to bed	12 (1.7)
Walking – training or mobilisation	10 (1.4)
Extrinsic factors^b^	8 (1.1)
Restraints that the patient attempts to get out of / over^c^	6 (0.8)
Not documented	300 (42.0)
Documentation regarding presence of anxiety/confusion before the fall, n (%)	
No	359 (50.3)
Yes	355 (49.7)

^a^Common area outside the ward (n = 4), outside the hospital building (n = 1), on leave but while admitted (n = 2), examination room (n = 1); ^b^ For example, inappropriate shoes, wet floors, knocked over, lack of wall handles in toilet / shower area, poor lighting, or objects that stand in the way and pose a tripping risk; ^c^ For example, bedrails or belt

There was no documentation in the records regarding fall-related activity, position, another person present and location in 42.0%, 36.8%, 36.0% and 20.7% of the falls (Table 2).

The sensitivity analyses indicated that the missing data were generally missing at random. However, the variable *another person present* showed a higher proportion of missing values among female patients and those younger than 65 years.

### Fall-preventive interventions

Nearly half of the patients (n = 449, 36.4%) had ≥ 1 intervention documented in the records, irrespective of fall risk. Of the 619 fall risk patients, according to DFRI, 360 (58.2%) had ≥ 1 documented intervention. Amongst the 590 fallers, 326 (55.3%) of the patients had ≥ 1 intervention and the corresponding figure for the 642 non-fallers was 123 (19.2%) (Fig 1).

**Fig 1:**
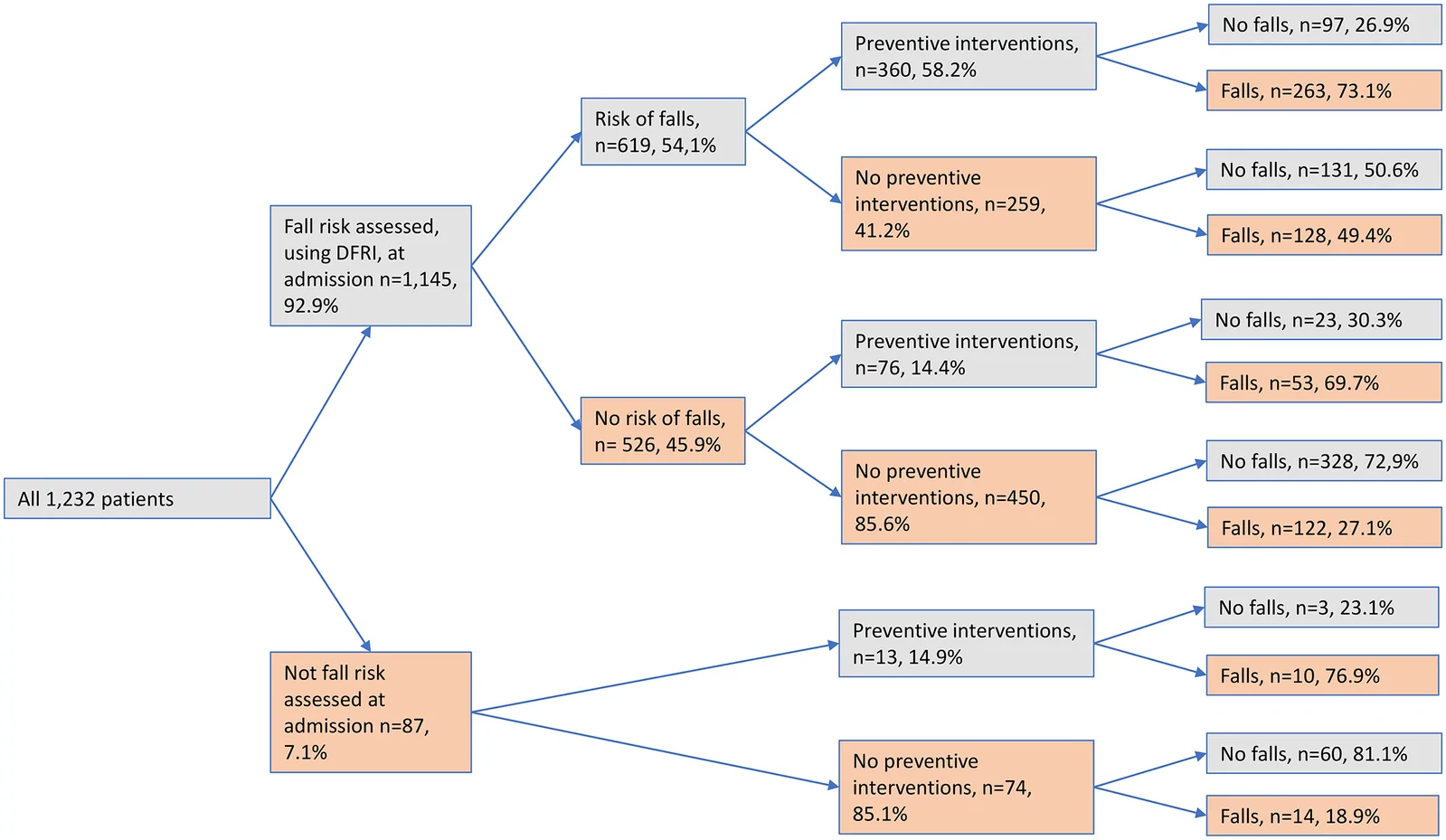
Patients with and without fall risk assessment at admission, fall-preventive interventions and fall outcomes. DFRI, Downton Fall Risk Index.

In total, 1,933 interventions were prescribed and documented, 370 (min-max, 1–11; median 3) in the non-fall group, 991 (min-max, 1–9; median 3) pre-fall for the fallers, and 572 (min-max, 1–11; median 3) post-fall for the fallers. Common interventions, for both the fallers and non-fallers, were adaptation of indoor environment, personal assistance during mobilisation and the use of non-slip socks or shoes (Table 3).

**Table 3:** Type of fall-preventive interventions, sorted on interventions in total.

Type of fall-preventive interventions^a^	Interventions in total, n (%)	Interventions in the non-fall group, n (%)	Interventions in the fall-group, pre-fall, n (%)	Interventions in the fall-group, post-fall, n (%)
Adaptation of indoor environment, e.g., “reach the buzzer” or remove tripping risks	303 (15.7)	50 (13.5)	163 (16.4)	90 (15.7)
Personal assistance during mobilisation	287 (14.8)	55 (14.9)	165 (16.6)	67 (11.7)
Non-slip socks / shoes during mobilisation	281 (14.5)	46 (12.4)	164 (16.5)	71 (12.4)
Observation/monitoring, e.g., extra supervision or alarm mats	283 (14.6)	52 (14.1)	116 (11.7)	115 (20.1)
Walking aids	243 (12.6)	60 (16.2)	119 (12.0)	64 (11.2)
Information to/educating patients	206 (10.7)	39 (10.5)	93 (9.4)	74 (12.9)
Restraints^b^	98 (5.1)	3 (0.8)	54 (5.4)	41 (7.2)
Personal support for personal care	91 (4.7)	33 (8.9)	34 (3.4)	24 (4.2)
Physiotherapist or dietitian assessment	54 (2.8)	10 (2.7)	38 (3.8)	6 (1.0)
Exercise/walking movement balance	27 (1.4)	5 (1.4)	17 (1.7)	5 (0.9)
Training in using aids	16 (0.8)	5 (1.4)	7 (0.7)	4 (0.7)
Information/teaching to relatives	12 (0.6)	1 (0.3)	7 (0.7)	4 (0.7)
Verbal guidance in personal care	12 (0.6)	2 (0.5)	7 (0.7)	3 (0.5)
Nutrition treatment	10 (0.5)	7 (1.9)	2 (0.2)	1 (0.2)
Training of dressing and undressing	6 (0.3)	2 (0.5)	4 (0.4)	0 (0.0)
Medication review	3 (0.2)	0 (0.0)	1 (0.1)	2 (0.3)
Continence training	1 (0.1)	0 (0.0)	0 (0.0)	1 (0.2)
Total	1,933 (100)	370 (100)	991 (100)	572 (100)

^a^The number of fall-preventive interventions is higher than the number of patients as one patient can have multiple fall-preventive interventions; ^b^ For example, bedrails or belt.

### Severity and type of harm, and response actions

Of the 714 falls, 429 (60.1%) had no documented harm. Among the 285 (39.9%) falls with harm, the majority (n = 263, 92.3%) were assessed as minor. The most common types of documented harm after the fall were pain, followed by wound and hematoma/swelling. Consultation or physical examination by a physician, check of vital functions and/or blood tests and X-rays were the most common response actions to the falls (Table 4).

**Table 4:** Severity and type of harm, and response actions taken in connection to the falls.

Variables		All falls, n (%)
Severity according to the NCC MERP scale		
C	An event that reached the patient but did not cause harm and no monitoring or intervention was carried out	198 (27.7)
D	An event that reached the patient and required monitoring or intervention to ensure that no harm occurred	231 (32.4)
E	Contributed or resulted in temporary harm	263 (36.8)
F	Contributed to or resulted in temporary harm that prolonged index hospitalisation, required additional in-patient admission(s) or extra out-patient care (known at discharge)	18 (2.5)
G	Contributed to or resulted in permanent harm	4 (0.6)
H	Required intervention necessary to sustain life within 60 minutes	0 (0.0)
I	Contributed to or resulted in the patient’s death	0 (0.0)
	Total	714
Type of harm^a^		
	Pain	128 (34.9)
	Wound (more severe than graze)	88 (24.0)
	Hematoma/swelling	82 (22.3)
	Graze	44 (12.0)
	Fracture	15 (4.1)
	Head injury/intracranial bleeding	4 (1.1)
	Confusion	4 (1.1)
	Sprain	2 (0.5)
Response actions^b^		
	Consultation or physical examination by a physician	295 (38.4)
	Check of vital functions, neurology status and/or blood tests^c^	186 (24.2)
	X-rays	131 (17.0)
	Wound care	82 (10.7)
	Increased surveillance	39 (5.1)
	Medication review and/or medications for pain/anxiety	17 (2.2)
	Allied health professional assessment and/or aids	15 (2.0)
	Surgery	4 (0.5)
	Total	769 (100)

NCC MERP, National Coordinating Council for Medication Error Reporting and Prevention

^a^The number of types of harm is higher than the number of patients with a harmful fall as one patient can have multiple types of harm. ^b^ The number of response actions is higher than the number of patients with a fall as one patient can have multiple types of actions taken. ^c^ By extra manual observations and/or by using monitors, or by transferring to a higher level of care.

### Timing of falls

Beyond the timing of falls presented in Table 2, the occurrence of falls ranged from admission day 1 to day 100. The second day of episode of care was the most common day for first falls (n = 150, 25.9%) and the majority of these occurred within the first three days on the index ward (n = 319, 55.0%).

The estimated median time to the first fall was 2 days (95% CI 1.0–2.0). There was a statistically significant difference in time-to-event between acutely admitted patients (2.0 days, 95% CI 1.0–2.0) and electively admitted patients (1.0 days, 95% CI 1.0–2.0, p < 0.001) (Fig 2).

**Fig 2:**
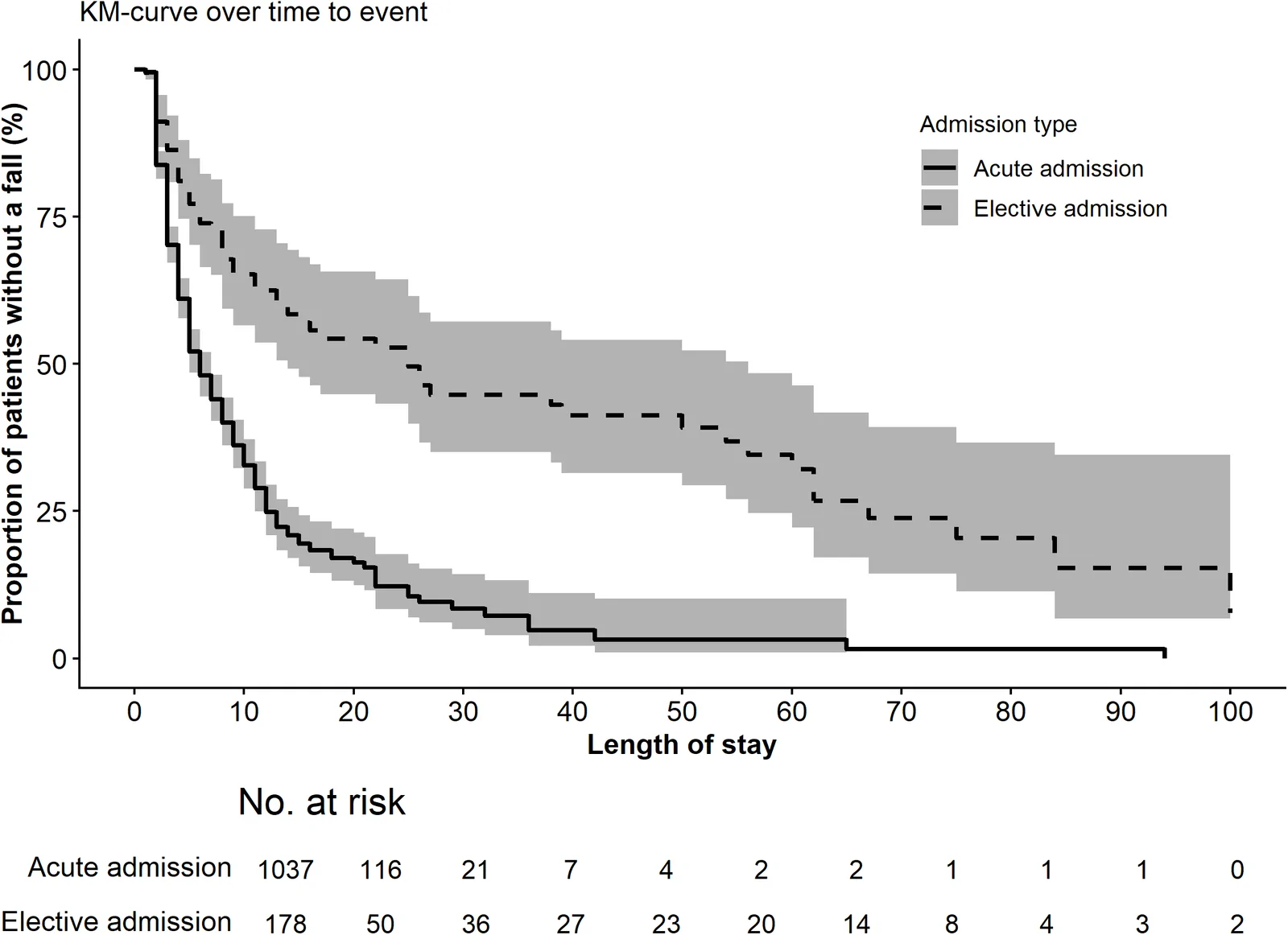
Proportion until a first fall over LOS until censored at discharge or first fall. Lines represent each admission type (starting with 1,037 for acute admissions and 178 for elective admissions). LOS, length of stay.

## Discussion

This study aimed to describe fall characteristics, consequences, and fall-preventive interventions across specialties. Falls typically occurred in the first days of admission, during night shifts and weekdays, in patient rooms and bathrooms, while walking, and were often unwitnessed. Most fall resulted in no documented harm; and when harm occurred, pain, wounds, and hematoma/swelling were the most common consequences. Environmental adaptation, personal assistance during movement, and non-slip socks or shoes were commonly used interventions.

### Occurrence of falls

In-hospital fall rates vary widely between studies [4]. As length of stay is a known risk factor [15,16], rates are often expressed per 1,000 patient days. Adjusted for the stratified sampling, our fall rate was 4.9 falls per 1,000 patient days. This is higher than in several studies [15,17–23], but lower than in others [5,24,25], which range from 0.41 [4] to 16.9 [5]. Our fall rate of 1.9% is lower than some studies [24–26] and comparable to others [27–29], possibly reflecting shorter lengths of stay, which can inflate rates per 1,000 patient days. Among fallers, 14.9% had multiple falls- slightly higher than reported by Heikkilä et al. [19], but lower than McKechnie et al*.* [24].

Comparing fall occurrence across studies is challenging due to variations in reported risk factors [4,30] and data collection methods. Many rely on incident reporting systems [26–29,31,32], which are prone to underreporting [33], reporting bias [34], and inconsistency, even when mandatory. Hibbert et al. [34] found that the record review method *Global Trigger Tool* identified over ten times more adverse events than incident reports. In the present study, the use of structured record review and the high proportion of elderly patients, a known fall risk factor, may partly explain the fall rates.

### Severity classification

Severity of falls is rated and classified using many different scales in previous studies, for example, mild, moderate and severe [21], low, medium or high [32], and minor, moderate, major or death [16]. Other scales used include no injury, minor, moderate, major or death [19], no injury, minor, moderate or severe [31], adverse event, serious adverse event or sentinel event [17], adverse event or non-adverse event [29], and serious or non-serious [28]. Different scales and varying definitions of the categories within the scales make comparison between studies challenging.

To quantify and classify fall severity, for example, the National Database of Nursing Quality Indicators (NDNQI) uses five standard categories, ranging from no injury to death [35,36]. The definitions in these categories mix harm types with response actions taken in connection to the fall. We used a modified version of the NCC MERP scale [14]. The scale is commonly applied in record review studies and quality improvement work on adverse events, both nationally and internationally, and with which some of the authors had previous experience. Reviewers found it challenging to assess the impact of less severe harm on length of stay (categories E vs. F) and to determine if more severe injuries caused permanent harm (category G). Assessing permanent harm is inherently challenging and is particularly difficult in prevalence studies like ours, where the design limits the ability to evaluate long-term outcomes. In retrospect, a fall-specific scale may have been easier to apply. The NDNQI scale was not used in the present study because it is not widely known or commonly applied in Sweden, and the authors were not aware of it at the time of data collection. Using NDNQI might have resulted in more falls being classified as moderate or major. On the other hand, NDNQI doesn’t include common outcomes such as pain or several fall-related response actions we identified. Therefore, even with prior awareness of the scale it may not have been considered a better or more user-friendly alternative.

The distinction between no-harm falls and minor injuries is often ambiguous and subject to interpretation. Perceptions of harm can influence, for example, the severity rating, fall injury rates and the type of harm outcomes.

### Occurrence and types of harm

In this study, fall severity varied and falls without harm were predominant. Harm of varying severity occurred in 40% of falls, and in line with previous studies reporting a wide range (1.6%–72.1%), most harmful falls were of low severity [4,27,32]. Compared to several studies [17–19,27,29,31,32,37,38], we observed a higher proportion of falls with harm, probably due to the inclusion of minor or short-term symptoms, such as patient-reported pain.

The most common types of harm were pain, wounds, and hematoma/swelling, which agrees with the findings of Heikkilä et al*.* [19]. However, the types reported vary across studies, and as no standardised categorisation exists this complicates comparisons. Some studies focus on injury location [22], others on severe outcomes like fractures or intracranial injuries [28], while others report only a few selected types [18,29]. In our study, fractures occurred in 4.1% of falls, within the reported range of 0% to 11.9% [17–19,21,22,28,29,39]. As a fall outcome, pain is often underreported. The distinction between a fall without an injury and a fall with a minor injury is not sharp and is therefore subject to assessment. The view of what constitutes harm may affect, for example, the severity rating, fall injury rates and the type of harm outcomes.

### No harm falls and fear of falling

Many studies have focused on harm and severe harm which is understandable due to patient suffering and extra costs incurred by the patients, the healthcare system and other services. In our study, 60% of the falls had no documented physical injury, consistent with the wide range (23.6%–98.4%) reported elsewhere [17–19,26,27,29,31,32,40]. However, no-harm falls may still involve factors that could potentially lead to harm in other circumstances. Thus, analysing both harmful and no-harm falls provides important opportunities for quality improvement and enhanced patient safety.

It is important to understand how a fall can affect the patient regardless of the severity of the fall. Falls can, for instance, lead to a fear of falling again, particularly among the elderly [7]. Although we did not capture such data, given its limited documentation in records, Baltes et al. [7] identified key coping strategies for older adults: managing risks, establishing safe surroundings and maintaining independence. If fear of falling is recognised, interventions can be introduced such as a combination of psychological support, physical exercise, and modifications in the home.

### Risk assessments

This study used the DFRI, which is the nationally recommended fall risk assessment tool for elderly patients. However, numerous tools are available, and Park [41] evaluated 26 fall risk assessment tools. The DFRI was found to be a relatively stable instrument with a pooled sensitivity of 0.84, but its pooled specificity of 0.26 was considered too low. Notably, the systematic review identified only two studies using DFRI, which were conducted in long-term care and community setting, respectively. Park [41] recommends using two tools, a view supported by Strini et al. [42] because of the multifactorial nature of fall risk. However, Morris et al. [43] found no evidence that scored tools reduce fall rates. The primary aim of such tools remains risk identification, not fall prevention [42].

There is limited evidence regarding fall risk assessment tools used to identify older hospitalised patients at risk of falls. The available evidence is of very low quality, and these tools have not demonstrated acceptable levels of sensitivity or specificity [44]. Therefore, the use of scored fall risk screening tools is not recommended [44–46]. A global initiative for fall prevention and management [45], recommends that all elderly patients be regarded as at risk of falling and the use of a multifactorial fall risk assessment tool should be considered for these patients.

In this study, compliance with fall risk assessment at admission using the DFRI was high, but follow-through with interventions based on assessment outcomes and clinical judgments were notably lower. Fall-risk individuals had higher DFRI scores compared to non-fallers; however, falls occurred among both high- and low-risk patients. Notably, one-third of the 526 patients assessed as low risk experienced a fall. This may partly be explained by the high number of elderly patients in the study cohort. These findings further emphasise the limitations of scored screening tools such as the DFRI when used in hospitalised patients, particularly in older populations. Like other fall risk assessment tools, the DFRI captures pre-defined risk factors at a single point in time, whereas fall risk in acutely ill older adults is dynamic and influenced by evolving clinical, cognitive, and environmental factors.

Risk assessment should not be limited to admission but repeated following health status changes or a fall [47], with corresponding updates to the fall prevention plan.

### Fall-preventive interventions

In many cases falls are preventable. Effective fall and harm prevention requires a comprehensive falls management strategy with a multidisciplinary approach including engaging patients. Interventions should be individualised to address each patient’s specific risk factors, as identified through a comprehensive falls assessment [44,45]. However, for the non-preventable falls, proactive interventions should be implemented to minimise fall-related harm [48].

Preventive interventions on the patient-level are seldom reported in non-intervention studies as ours. While various interventions were documented, they were underutilised relative to the fall risk assessment score. Just over half of patients identified as at risk by DFRI had a documented intervention prior to the fall, compared to 88.7% in the study by Costantinou and Spencer [49]. That study also reported underuse of preventive resources and interventions. Furthermore, they present some interventions such as low beds, floor mats and hourly rounding.

Nearly three-quarters of those with interventions in our study still fell, while at-risk patients without interventions fell less frequently, raising questions about intervention effectiveness [50]. Although differences in the use of fall-preventive interventions were observed both between the fall and non-fall groups and the low-risk and at-risk groups, the impact of these differences and interventions remains difficult to interpret. Fall prevention in clinical practice is inherently multifactorial which makes it challenging to isolate the impact of any single preventive intervention. This finding is not unique to the present study. Our findings highlight the complexity of evaluating fall-prevention interventions in clinical settings. A recent systematic Cochrane review [50] states that fall prevention in hospitals is highly challenging and therefore warrants further research on the effectiveness of all intervention types aimed at preventing falls in hospitals. Despite the large number of trials included in the review, the conclusions for many intervention types remain uncertain.

Patient and staff education can reduce fall rates and risk [43,50]. In the present study, patient education was part of the documented fall-preventive interventions, though it remains an area for improvement. Patients often underestimate their fall risk and overestimate their mobility [51–53], highlighting the need for collaborative risk assessment [51]. Heng et al. [54] conclude that education-based programs can reduce falls by increasing patients’ knowledge and awareness of their fall risk, empowering self-management.

Non-slip socks and shoes during mobilisation were part of the most common preventive interventions in our study. The evidence supporting the use of non-slip socks to prevent falls in hospitals is not conclusive. These are often part of multi-component interventions, and it is therefore not easy to determine the effectiveness of the non-slip socks alone [55,56]. However, they do not constitute adequate footwear to prevent falls [55].

In our study, multiple fall-preventive interventions per patient were common. Multifactorial interventions, where interventions are based on a fall risk assessment, can probably reduce hospital-fall rates [43,50]. Zegers et al. [57] found in an umbrella review that the fall rate was lowered when using multi-component interventions. A global initiative [45] recommends implementing individualised fall prevention strategies, whether single- or multi-domain, for all older hospitalised patients and at-risk younger individuals, based on identified risk factors, behaviours, or situations.

Evidence-based hospital falls guidelines exist, but their implementation and compliance remain limited within healthcare [46,50,58]. The effectiveness of implementing fall-preventive interventions among hospitalised adults is variable. A systematic review concluded that the operationalisation of implementation strategies for fall-preventive interventions is often insufficiently described, limiting the reproducibility of implementation efforts. Consequently, clinicians may face challenges in selecting evidence-based strategies for implementation, while researchers are hindered in examining the relationships between implementation strategies and outcomes [58]. Although fall-prevention bundles may reduce the risk of falls, their impact on overall fall rates remains uncertain [50].

### Response actions to the fall

The most common response actions taken included physician consultations or examinations, check of vital functions, neurology status and/or blood tests and X-rays. Few studies detail immediate responses to falls [21,39]. In direct relation to a fall, the recommendation is to perform an examination to evaluate the occurrence and severity of any harm, carry out relevant treatment and care, and continue observing for later onset harm that may not be evident immediately after the fall. Furthermore, identification of factors that may have contributed to the fall is needed. Collaboration with the patient and the multiprofessional team is essential to determine relevant interventions [48].

### Timing

Falls occur 24/7. Our results showed that most of the falls occurred during the night shifts followed by morning shifts. Night shifts being the shift with most falls are confirmed by several other studies [17,18,23,25,28,31,49], though others [19,24] have reported morning shifts as the peak period.

Nighttime falls may be linked to reduced staffing levels. In the present study the night shifts are somewhat longer and have a lower nurse-to-patient ratio. Studies show that organisational factors such as nurse staffing levels are associated with inpatient fall rates. Greater registered nurse hours per patient day [59–61] and higher perceived staffing adequacy are associated with lower fall rates [59]. Furthermore, an increase in nursing time per patient during the night is associated with lower fall rates [62]. Improved staffing may enable closer patient monitoring and timely interventions. In addition to staffing levels, higher staff turnover rates are associated with increased patient falls [63]. Furthermore, nursing units with strong safety climate and working conditions and lower missed care are associated with lower fall rates [64]. Although staffing levels and shift workload were not included in our study, these factors, especially during night shifts, may have influenced both the occurrence of falls and the documentation of fall outcomes.

Additional reasons can be less frequent patient checks or the exclusion of toileting to enhance patient sleep. Hourly rounding to proactively anticipate needs using systematic checks may decrease falls in hospitals. However, because it may disrupt sleep, efforts should be made to mitigate potential unintended negative effects [48]. Furthermore, the patients express the importance of mobilising to the bathroom quickly and independently to avoid incontinence [65]. It may be assumed that patients potentially rise before staff assistance arrives, especially if they overestimate their mobility [49,66]. Hospitalised patients who visit the bathroom alone at night are more vulnerable to falls as they are exposed to unfamiliar surroundings and poor lighting [49]. The physical environment includes several modifiable risk factors for falls which may limit patients’ ability to move safely. Factors that should therefore be considered include appropriate flooring, adequate lighting (for example, night lighting), suitable furniture, safe and functional equipment, and supportive features such as sturdy handholds and handrails [48].

The median time to the first fall was 2 days, with elective patients falling earlier than those admitted acutely. We could not find any other studies to compare the timing in relation to the type of admission. In both groups, most falls occurred within the first 3 days. Our median time to fall is earlier compared to other studies, where a range was found between 3–27 days [18,19,25,28,38,49,67]. The length of stay itself increases the risk of falls [15,16]. The fallers in our study had a longer length of stay than non-fallers but this can be partly due to other reasons besides the fall.

### Documentation

In similarity to other studies [19,32], we found a high number of missed documentations in relation to, for example, position and fall-related activities. Like us, Yasan et al. [68] identified a gap between recognising high fall-risk patients, and the documentation and execution of fall-preventive interventions. Pernes et al. [69] state that this contravenes the recommendations for documenting falls to measure for their recurrence from a preventive perspective. Based on their scoping review, the authors recommend that fall documentation should include the patient’s identification, the time and location of the fall, the mechanism of the incident, the activity being performed at the time, the resulting consequences, and the actions taken afterward. Schmitz et al. [70] suggest that the use of a structured post-fall template within the patient record may help standardise and improve the quality and completeness of information documented following a fall.

### Strengths and limitations

This study has both strengths and limitations. One of the strengths is the comprehensive data collection from all health professionals’ entries, allowing detailed fall analysis. The data reliability is supported by the very good kappa value [13]. In addition, the setting is a large acute public hospital serving an extensive catchment area. The patients were treated in different clinical departments encompassing both acute and planned admissions, reflecting the national standard of care.

The present study has some limitations. Firstly, its retrospective design and single centre setting limit generalisability. Secondly, reliance on record review captured only documented falls, introducing potential information bias and underestimation of the fall rate. For some variables related to fall demographics, a substantial amount of data was missing. Due to the absence of information for these variables in the records, there is a risk that unmeasured characteristics may have influenced results. The sensitivity analyses showed that the missing data were largely missing at random. Thirdly, patients were not followed post-discharge, potentially affecting outcomes such as severity assessment.

## Conclusions

Understanding the characteristics and consequences of falls forms the foundation of effective fall prevention and safety strategies, thereby advancing care. This study provides new insights into the timing, context, and outcomes of in-hospital falls. There are still patients who fall despite fall-preventive interventions, highlighting critical gaps in fall prevention practice. More personalised, evidence-based interventions targeting modifiable risk factors, particularly during the first days of hospitalisation and night shifts, can potentially reduce the risk of in-hospital falls and improve patient outcomes. Additional research is required to determine the effectiveness of interventions designed to prevent falls in hospital settings.
